# The impact of postoperative EGFR-TKIs treatment on residual GGO lesions after resection for lung cancer

**DOI:** 10.1038/s41392-020-00452-9

**Published:** 2021-02-21

**Authors:** Bo Cheng, Caichen Li, Yi Zhao, Jianfu Li, Shan Xiong, Hengrui Liang, Zhichao Liu, Wenchuang Zeng, Wenhua Liang, Jianxing He

**Affiliations:** 1 Department of Thoracic Surgery and Oncology, the First Affiliated Hospital of Guangzhou Medical University, State Key Laboratory of Respiratory Disease & National Clinical Research Center for Respiratory Disease, Guangzhou, 510120 China; 2 Department of Thoracic Surgery, Shanghai Chest Hospital, Shanghai Jiao Tong University, Shanghai, China

**Keywords:** Lung cancer, Cancer genetics, Drug development

**Dear Editor**,

In recent years, the incidence of multifocal lung cancer is increasing, called multiple primary lung cancer (MPLC). Synchronous MPLC (sMPLC) is defined by multiple malignant lesions occurring at the same time.^1^ In clinical practice, it was always extremely difficult to remove all lesions simultaneously for sMPLC patients. However, unresected GGO lesions after primary surgery frequently caught the risk of progression. Therefore, an effective postoperative treatment was necessary for these patients to prevent recurrence or progression of disease, especially for these patients who were unsuitable or unwilling to receive one more operation.

In multiple primary lung adenocarcinomas, there is a high proportion of patients harboring EGFR mutations, and most lesions present as multiple ground-glass opacity (GGO) in imaging examinations.^2^ According to the Chinese Society of Clinical Oncology (CSCO) guideline, EGFR-TKIs are recommended to be used as adjuvant treatment after surgery of EGFR-mutated early-stage NSCLC. However, it is unclear whether postoperative EGFR-TKIs show efficacy on residual early-stage GGO lesions in patients who undergo resection of their main lesion(s) and harboring EGFR mutations. Therefore, the objective of this study was to observe whether postoperative EGFR-TKIs show efficacy on unresected persistent GGO lesions in sMPLC patients with EGFR mutations.

In this study, we retrieved patients who were considered to have MPLC and underwent resection of at least one EGFR-mutated lesion between 2014 and 2018 from the database. Included patients should have one or more residual malignant ground-glass nodules (<3 cm) whose malignancy were confirmed by both a radiologist and a thoracic surgeon (LHR and XS) (Supplementary Fig 1). The definition for MPLC is based on previously published clinical criteria (Supplementary Table 1).^3^ Treatment group included sMPLC patients treated with postoperative EGFR-TKIs, involving Gefitinib, Erlotinib, Icotinib, Afatinib and Osimertinib. Patients not treated with EGFR-TKIs after surgery were set as controls.

Low-dose thin-slice CT (1 mm thickness) was used for imaging examination in all patients, once every 2–3 months. As the largest diameter of most lesions was less than 1 cm, the definition of evaluable lesion of RECIST 1.1 criteria (1 cm or more in largest diameter) could not be met.^4^ Therefore, we expanded the lower limit of the diameter to 4 mm, as the thin-slice CT scan can precisely detect the minor changes in size by 2 mm. The definition of objective response rate (ORR) were identical to RECIST 1.1. In addition, the response rate (RR) of EFGR-TKIs was herein defined as the ratio of the patients/lesions with any diametrical reduction shown in CT scans to the whole cohorts of patients/lesions, respectively. Some specific section of the methods in this research such as the definition for MPLC, the set of some subgroup and the statistical analyses were placed in supplementary materials. Meanwhile, we measured the consistency of gene mutation between multiple lesions when multiple different lesions (≥2) were resected in a same patient. This study was approved by the ethics committee of The First Affiliated Hospital of Guangzhou Medical University and the informed consent of patients was waived.

A total of 143 eligible patients with sMPLC were included. Among them, 66 patients with 134 unresected malignant GGO lesions were treated with postoperative EGFR-TKIs, while the other 77 patients received observation only. Baseline information of these 66 patients treated with EGFR-TKIs is shown in supplementary Table 2.

## Lesion-oriented response rate of EGFR-TKIs

**Fig. 1:**
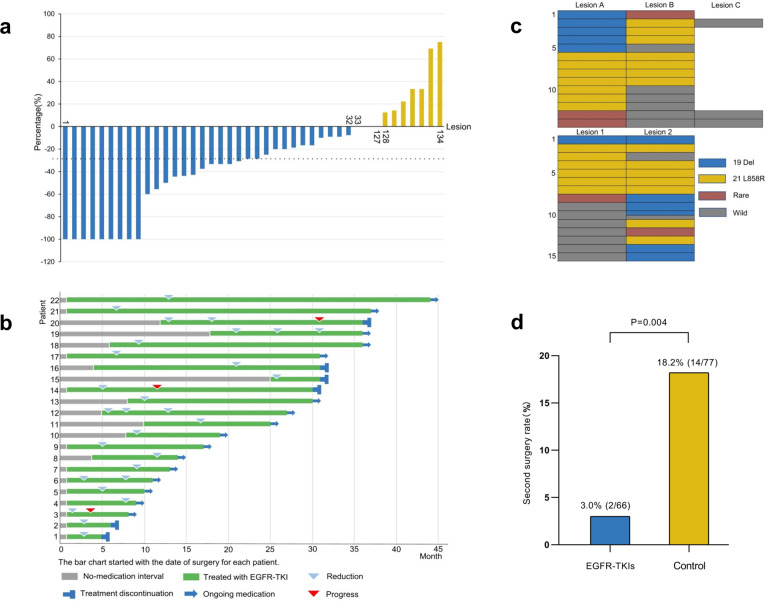
**a** The changes in size of unresected GGO lesions during the medication. **b** The information of drug use was recorded in 22 patients with reduction in size of lesions. **c** The distribution of EGFR mutations in 29 patients with multiple lesions resected. The upper and lower tables show the types of mutation in patients who underwent one and two operations, respectively. The Lesion A, B and C represent different lesions resected in one same operation, while the Lesion 1 and 2 represent the lesions resected in the first and second operation. Meanwhile, for patients who underwent two operations, different colors were filled in the same table cell when multiple lesions were removed in one operation. **d** The second surgery rate of unresected lesions in the EGFR-TKIs treatment group and control group

We recorded the changes in size of 134 lesions in 66 patients treated with postoperative EGFR-TKIs. In terms of lesions, the response rate (RR) is 23.9% (32/134). As illustrated in the waterfall plot (Fig. 1a), 32 lesions reduced, 7 lesions increased, and 95 lesions had no change in size.

The RR and ORR in different subgroups were presented in supplementary Table 3. There were significant differences in two subgroups: (1) the diameter of lesions ≥8 mm or <8 mm (42.9 vs 15.2%, *P* < 0.01) and (2) mixed or pure-GGO lesions (34.0 vs 17.9%, *P* = 0.03). Based on RECIST 1.1 criteria, the objective response rate of EGFR-TKIs is 14.9% (20/134), and the statistical results of these subgroups were similar to the RR. The drug use was recorded in the 22 patients with reduction in size of lesions (Fig. 1b).

## Patient-oriented response rate of EGFR-TKIs

In terms of patients, 22 patients had reduction in size of any lesion and the response rate of patients treated with postoperative EGFR-TKIs was 33.3% (22/66). Supplementary Fig. 2 shows typical changes in CT scans of patients who had a significant response to EGFR-TKIs.

Of the different subgroups, there were significant differences between the patients with maximum diameter of residual GGO lesions ≥8 mm or <8 mm (58.6 vs 13.5%, *P* < 0.01), patients with more than 2(max 5) or 1–2 remaining malignant GGO lesions (54.2 vs 21.4%, *P* < 0.01), and patients with major lesions at stage III or stage I-II (61.5 vs 26.4%, *P* = 0.02). According to RECIST 1.1 criteria, the objective response rate of EGFR-TKIs is 19.7% (13/66), and the statistical results of each subgroup was similar to that of the RR. For comparison, we observed 77 patients who did not receive EGFR-TKIs after surgery. 9.1% (7/77) had a reduction in size of residual GGO lesions, which was significantly lower than the EGFR-TKIs treatment group (*P* < 0.01).

## Independent predictors for response of EGFR-TKIs

As shown in supplementary Table 3, a multivariable logistic regression model was created using some following covariates. These covariates were chosen based on the results of Chi-Square test. The efficacy of EGFR-TKIs was associated with a greater number of residual lesions (OR = 4.33, *P* < 0.01), larger diameter of the maximal residual lesion (OR = 9.07, *P* < 0.01), and higher stage of primary lesions (OR = 4.46, *P* = 0.02). In addition, in terms of the characteristics of lesions, lesions with mixed component (OR = 2.37, *P* = 0.04) and larger diameter of residual lesions (OR = 4.18, *P* < 0.01), were independent predictors for EGFR-TKIs efficacy.

Among 144 patients with postoperatively residual malignant GGO lesions, 29 patients underwent surgical resection of multiple lesions (2 or 3) and genetic testing. Interestingly, the consistency rate of 19 Del mutation was significantly lower than that of 21 L858R mutation (3.4 vs 31.0%, *P* = 0.03). Gene mutation types of these 29 patients are illustrated in Fig. 1c. Summary of cases sequentially using different EGFR-TKIs or surgery were recorded in Supplementary Table 4 and Supplementary Table 5. It’s worth noting that the rate of second resection of residual lesions was significantly lower in the EGFR-TKIs treatment group compared to the control group (3.0 vs 18.2%, *P* = 0.004) (Fig. 1d).

To our knowledge, this study is the first study to observe the impact of postoperative EGFR-TKIs treatment on unresected GGO lesions in sMPLC patients after the surgery of major lesion(s) harboring EGFR-mutation. In our study, EGFR-TKIs show efficacy on these unresected GGO lesions but was limited. Notably, the response rate is lower compared with advanced stage patients in historical controls,^5^ this could be attributed to the genetic heterogeneity among lesions. However, patients with more aggressive phenotypes have significant benefit from EGFR-TKIs in this study. Therefore, when considering postoperative EGFR-TKIs treatment for multifocal lung cancer, it is critical to determine the mutation status of each residual lesion.

In summary, postoperative EGFR-TKIs treatment show activity on persistent GGO lesions remaining after surgery and for these patients there was significant benefit if they presented with TNM stage III, had more than 2 remaining lesions, mixed component lesions, or the diameter of residual lesions ≥8 mm.

## Supplementary information

A read-me for supplementary materials

Supplementary Materials

## Data Availability

All data and materials are available to the researchers once published.
