# Supplementary material for: The impact of postoperative EGFR-TKIs treatment on residual GGO lesions after resection for lung cancer
Source: Signal Transduct Target Ther. 2021 Feb 21;6:73. doi: 10.1038/s41392-020-00452-9 (PMC7897326; doi:10.1038/s41392-020-00452-9)
Supplement: Supplementary file 1 — A read-me for supplementary materials [file 41392_2020_452_MOESM1_ESM.docx]

**A ‘read-me’ for supplementary materials**

Our supplementary file included the following information：

(1) Some specific sections of the methods in this research such as the definition for MPLC (**supplementary Table 1**), the set of some subgroups and the statistical analyses were placed in supplementary materials. Besides, we have added detailed explanations of **Fig 1**(containing **Fig.1a, 1b, 1c and 1d**) and the outcomes like response rate of patients into the supplementary materials.

(2) The process for retrieving patients who met the inclusion criteria of our study, as shown in **supplementary Fig 1**.

(3) CT changes of lesions when EGFR-TKIs was effective on patients, as shown in **supplementary Fig 2**.

(4) The baseline information of patients in the EGFR-TKIs treatment group and control group, as shown in **supplementary Table 2**.

(5) Clinical characteristics and the response rate of enrolled patients (and lesions), as shown in **supplementary Table 3**.

(6) Case summary of patients treated with two kinds of EGFR-TKIs in succession (**supplementary Table 4**) and patients who underwent two operations and received postoperative EGFR-TKIs treatment (**supplementary Table 5**).
