# Supplementary material for: The impact of postoperative EGFR-TKIs treatment on residual GGO lesions after resection for lung cancer
Source: Signal Transduct Target Ther. 2021 Feb 21;6:73. doi: 10.1038/s41392-020-00452-9 (PMC7897326; doi:10.1038/s41392-020-00452-9)
Supplement: Supplementary file 2 — Supplementary Materials [file 41392_2020_452_MOESM2_ESM.docx]

**Supplementary Materials**


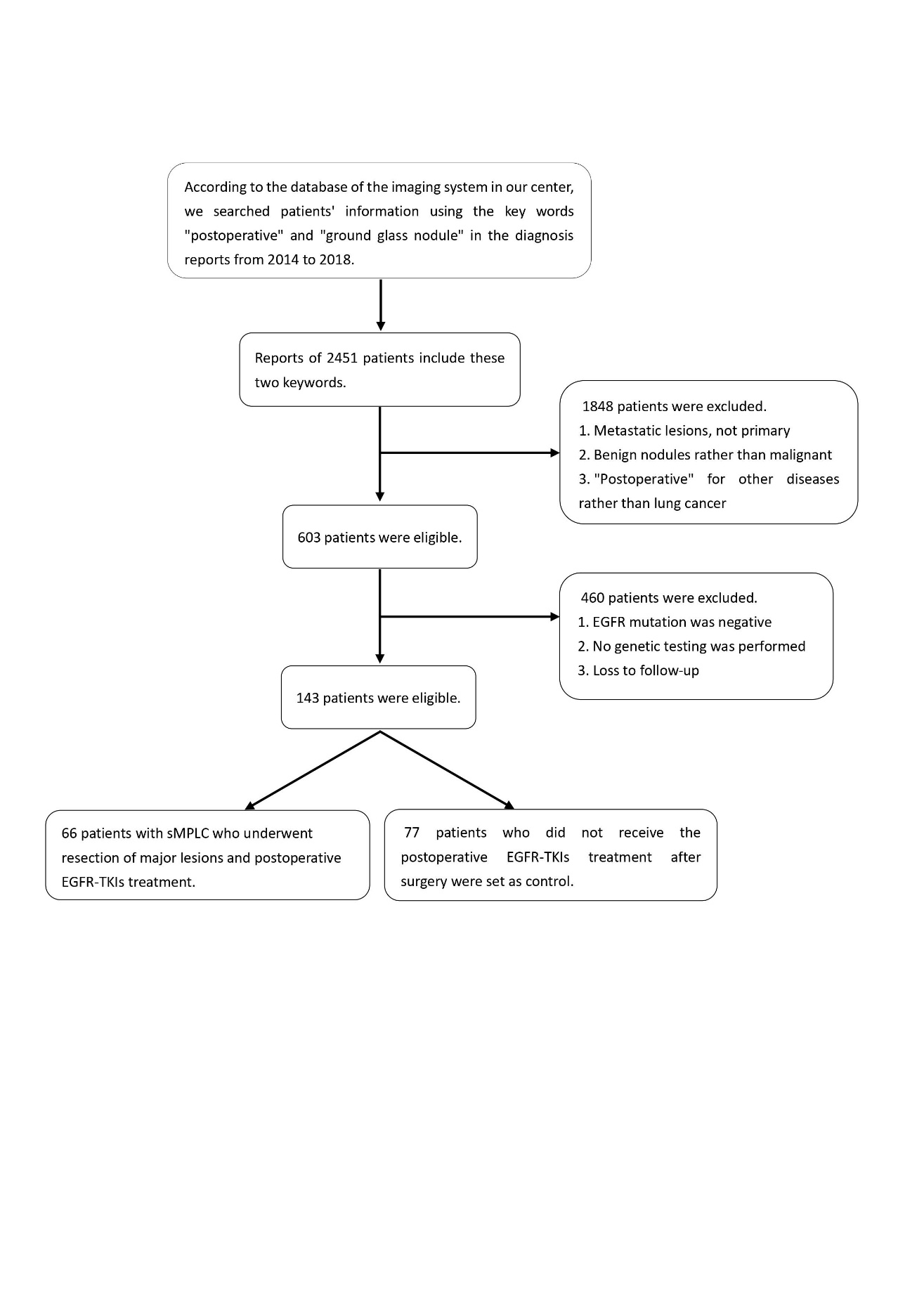
**Figure S1.** **Screening** **process for eligible patients.**

**Figure S2.**

**CT changes of lesions when EGFR-TKI was effective on patients.**

**Patient 1**


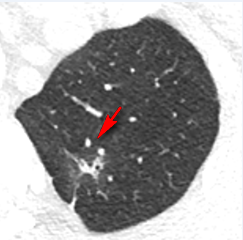
**
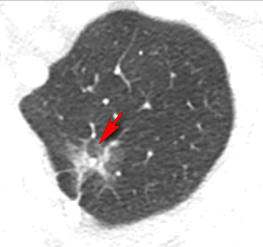
**

**Patient
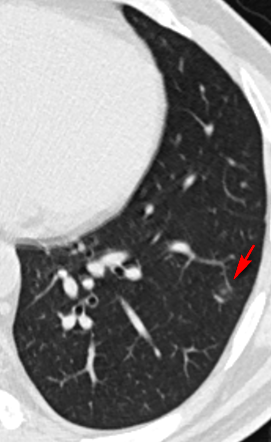
2**

**
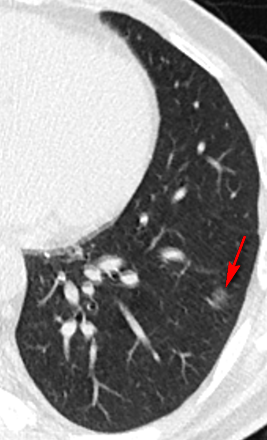
**

**Table S1.**

**The inclusion criteria for sMPLC patients were based on the clinical criteria published by Frank c. Detterbeck et al in 2016, as shown in this following table.**

| Clinical Criteria for Separate versus Related Pulmonary Tumors |
| --- |
| **Clinical criteria^*^** |
| Tumors may be considered separate primary tumors if They are clearly of a different histologic type (e.g., squamous carcinoma and adenocarcinoma). |
|  |
| Tumors may be considered to be arising from a single tumor source if Matching breakpoints are identified by comparative genomic hybridization. |
|  |
| Relative arguments that favor separate tumors: Different radiographic appearance or metabolic uptake Different pattern of biomarkers (driver gene mutation) Different rates of growth (if previous imaging is available) Absence of nodal or systemic metastases |
|  |
| Relative arguments that favor a single tumor source: The same radiographic appearance Similar growth patterns (if previous imaging is available) Significant nodal or systemic metastases The same biomarker pattern (and same histotype) |
|  |

^*^ Note that a comprehensive histologic assessment is not included in clinical staging, as it requires that the entire specimen has been resected.

**Table S2**

**The baseline information of patients in the EGFR-TKIs treatment group and control group.**

| Characteristics | Type | EGFR-TKIs | Control |
| --- | --- | --- | --- |
|  |  | (N=66) | (N=77) |
| Sex | Male | 21 (31.8) | 23 (29.9%) |
|  | Female | 45 (68.2) | 54 (70.1%) |
| Age | ≥50 | 59 (89.4) | 57 (74.0%) |
|  | <50 | 7 (10.6) | 20 (26.0%) |
| Mutation ^a^ | 21 L858R | 36 (56.3) | 41 (53.2%) |
|  | 19 DEL | 21 (32.8) | 29 (37.7%) |
|  | Rare | 7 (10.9) | 7 (9.1%) |
| Smoking History | Yes | 8 (12.1) | 12 (15.6%) |
|  | No | 58 (87.9) | 65 (84.4%) |
| Co-morbidities ^b^ | Yes | 22 (33.3) | 14 (18.2%) |
|  | No | 44 (66.7) | 63 (81.8%) |
| FEV1/FVC ^c^ | ≥0.7 | 57 (89.1) | 68 (93.2%) |
|  | <0.7 | 7 (10.9) | 5 (6.8%) |
| Pathology ^d^ | AIS | 1 (1.5) | 3 (3.9%) |
|  | MIA | 5 (7.6) | 10 (13.0%) |
|  | IA | 60 (90.9) | 64 (83.1%) |
| TNM Stage ^e^ | I | 39 (59.1) | 72 (93.5%) |
|  | II | 14 (21.2) | 2 (2.6%) |
|  | III | 13 (19.7) | 3 (3.9%) |
| Number of Remaining Lesions | 1 | 30 (45.5) | 36 (46.8) |
|  | 2 | 12 (18.2) | 21 (27.3) |
|  | 3 | 17 (25.8) | 12 (15.6) |
|  | 4 | 5 (7.6) | 5 (6.5) |
|  | 5 | 2 (3.0) | 3 (3.9) |
| Maximum Diameter of Remaining Lesions | ≥8mm | 29 (43.9) | 26 (33.8%) |
|  | <8mm | 37 (56.1) | 51 (66.2%) |
| Follow-up ^f^  (Months) | Median | 30 | 27 |
|  | Range | 6-58 | 7-58 |
| EGFR-TKI ^g^ | Gefitinib | 20 (30.3) | -- |
|  | Erlotinib | 16 (24.2) | -- |
|  | Icotinib | 17 (25.8) | -- |
|  | Afatinib | 2 (3.0) | -- |
|  | Osimertinib | 1 (1.5) | -- |

^a^ Two patients in the EGFR-TKIs treatment group had both 21 L858R and 19 Del mutation in the main lesion, and were not included in any mutation group. (21 L858R=exon 21 L858R mutation, 19 DEL=exon 19 deletion mutation, Rare=rare mutations)

^b^ Co-morbidities include diabetes, hypertension and coronary artery disease.

^c^ Six patients' results of pulmonary function test were not obtained. (Two in EGFR-TKIs treatment group and four in control group; FEV1: Forced expiratory volume in 1 second, FVC: Forced vital capacity)

^d^ AIS: Adenocarcinoma in situ MIA: Minimally invasive adenocarcinoma IA: Invasive adenocarcinoma

^e^ TNM classification: T for ‘Primary tumor’, N for ‘Regional lymph node involvement’, M for ‘Distant metastasis’.

^f^ Follow-up period of patients in these two groups was calculated from the date of operation.

^g^ Ten patients (15.2%) were treated with two types of EGFR-TKI successively, including Gefitinib and Osimertinib、Erlotinib and Osimertinib、Icotinib and Osimertinib、Erlotinib and Gefitinib、Icotinib and Gefitinib、Gefitinib and Icotinib.

**Table S3.**

**Clinical characteristics and response rate of enrolled patients (and lesions)**

| Characteristics | Groups | Response Rate (%) | **P Value** | Objective Response Rate (%) | **P Value** |
| --- | --- | --- | --- | --- | --- |
| **Lesions** | | | | | |
| Overall | | 23.9 (32/134) |  | 14.9 (20/134) |  |
| Diameter | ≥8mm | 42.9 (18/42) | **<0.01** | 26.2 (11/42) | **0.01** |
|  | <8mm | 15.2 (14/92) |  | 9.8 (9/92) |  |
| Constituent | Mixed | 34.0 (17/50) | **0.03** | 20.0 (10/50) | **0.20** |
|  | Pure | 17.9 (15/84) |  | 11.9 (10/84) |  |
| **Patients** | | | | | |
| Overall | | 33.3 (22/66) |  | 19.7 (13/66) |  |
| Sex | Male | 38.1 (8/21) | **0.58** | 23.8 (5/21) | **0.57** |
|  | Female | 31.1 (14/45) |  | 17.8 (8/45) |  |
| Smoking History | Yes | 37.5 (3/8) | **0.79** | 12.5 (1/8) | **0.59** |
|  | No | 32.8 (19/58) |  | 20.7 (12/58) |  |
| Co-morbidities | Yes | 45.5 (10/22) | **0.14** | 27.3 (6/22) | **0.27** |
|  | No | 27.3 (12/44) |  | 15.9 (7/44) |  |
| Mutation ^a^ | 21 L858R | 38.9 (14/36) | **0.39** | 25.0 (9/36) | **0.34** |
|  | 19 DEL | 28.6 (6/21) |  | 9.5 (2/21) |  |
|  | Rare | 14.3 (1/7) |  | 14.3 (1/7) |  |
| EGFR-TKI ^b^ | Gefitinib | 50.0 (10/20) | **0.42** | 30.0 (6/20) | **0.74** |
|  | Erlotinib | 31.3 (5/16) |  | 18.8 (3/16) |  |
|  | Icotinib | 29.4 (5/17) |  | 11.8 (2/17) |  |
|  | Afatinib | 0 (0/2) |  | 0 (0/2) |  |
|  | Osimertini | 0 (0/1) |  | 0 (0/1) |  |
| Stage | I-II | 26.4 (14/53) | **0.02** | 17.0 (9/53) | **0.26** |
|  | III | 61.5 (8/13) |  | 30.8 (4/13) |  |
| Remaining Lesions | 1-2 | 21.4 (9/42) | **<0.01** | 9.3 (4/43) | **<0.01** |
|  | 3-5 | 54.2 (13/24) |  | 39.1 (9/23) |  |
| Diameter ^c^ | <8mm | 13.5 (5/37) | **<0.01** | 5.4 (2/37) | **<0.01** |
|  | ≥8mm | 58.6 (17/29) |  | 37.9 (11/29) |  |

^a^ Two of the 66 patients had both 21 L858R and 19 Del mutation in the main lesion, and were not included in any mutation group.

^b^ Ten patients received two kinds of EGFR-TKIs treatment in succession, and were not included in this analysis.

^c^ We measured the diameter of the remaining lesions and recorded the diameter of the largest lesion of patients.

**Table S4.**

**Case summary of patients treated with two kinds of EGFR-TKIs in succession.**

| Patient | Gender | Age | | Stage | | Mutation | First  EGFR-TKI | Time  (month) | | Size or density change | Second  EGFR-TKI | Time  (month) | Size or density change |
| --- | --- | --- | --- | --- | --- | --- | --- | --- | --- | --- | --- | --- | --- |
| 1 | Female | 30 | I | | Rare | | Gefitinib | 2 | No | | Osimertinib | 2 | No |
| 2 | Female | 45 | IV | | 19 Del | | Gefitinib | 27 | No | | Osimertinib | 1 | No |
| 3 | Female | 65 | III | | Rare | | Erlotinib | 19 | No | | Osimertinib | 12 | No |
| 4 | Female | 59 | II | | 21 L858R | | Icotinib | 15 | No | | Osimertinib | 3 | No |
| 5 | Female | 72 | I | | 21 L858R | | Erlotinib | 2 | No | | Gefitinib | 4 | No |
| 6 | Female | 56 | III | | 21 L858R | | Icotinib | 2 | No | | Gefitinib | 12 | No |
| 7 | Female | 60 | I | | 19 Del | | Icotinib | 11 | No | | Gefitinib | 7 | No |
| 8 | Male | 70 | II | | Rare | | Gefitinib | 7 | No | | Icotinib | 1 | No |
| 9 | Female | 68 | III | | Rare | | Gefitinib | 4 | No | | Icotinib | 3 | Reduced |
| 10 | Female | 76 | I | | 21 L858R | | Gefitinib | 21 | Reduced | | Icotinib | 8 | No |

**Table S5.**

**Patients who underwent two operations and received postoperative EGFR-TKIs treatment.**

| Patient | EGFR Mutation of 1th Lesion | Stage | EGFR-TKI | Interval  (Month) | EGFR Mutation of 2nd Lesion | EGFR-TKI |
| --- | --- | --- | --- | --- | --- | --- |
| 1 | Wild type | I | No | 2 | 19 Del | Icotinib |
| 2 | 21 L858R | I | No | 1 | 21 L858R | Erlotinib |
| 3 | 21 L858R | I | Icotinib | 49 | No Detection | No |
| 4 | 21 L858R | I | No | 35 | 21 L858R | Osimertinib |
| 5 | Wild type | I | No | 4 | 19 Del | Gefitinib |
| 6 | 19 Del | I | No | 14 | No Detection | Icotinib |
| 7 | 21 L858R | I | No | 12 | No Detection | Icotinib |
| 8 | 21 L858R | I | Erlotinib | 3 | No Detection | No |

**Some detailed description for the methods part in this study**

***Eligible Patients***

Since July 2014, a prospective cohort of patients who received pulmonary resection at our center has been established, with detail information and regular follow-up. In this study, we retrieved patients who were considered to have multiple primary lung cancer and underwent resection of at least one EGFR-mutated lesion between 2014 and 2018 from the database. Included patients should have one or more residual malignant ground-glass nodules (< 3cm) whose malignancy were confirmed by both a radiologist and a thoracic surgeon (LHR and XS). Only patients whose resected main lesion(s) harbored an EGFR mutation were included for this analysis. Gene detection of enrolled patients includes two methods: The amplification-refractory mutation system (ARMS) and the next-generation sequencing (NGS). The flow chart is described as Figure 1. ***Definition for Multiple Primary Lung Cancer***

The definition for MPLC is based on previously published clinical criteria (Table S1).[1] To determine which sMPLC patients met our inclusion criteria, we analyzed all available information at our multidisciplinary team meetings. In this study, the TNM categories of resected lesions were uniformly based on the eighth edition of the TNM Classification for Lung Cancer of the International Association for the Study of Lung Cancer (IASLC).[2]

***Interventions and Controls***

Treatment group included patients treated with EGFR-TKIs, involving first-generation drug Gefitinib (250mg), Erlotinib (100mg, 150mg), Icotinib (125mg), second-generation drug Afatinib (40mg) and third-generation drug Osimertinib (80mg). Patients not treated with EGFR-TKIs after surgery were set as controls.

***Outcome Measures***

Low-dose thin-slice CT (1mm thickness) was used for imaging examination in all patients, once every 2-3 months. The size changes of residual GGO lesions in computed tomography (CT) scan after treatment were evaluated. We considered the reduction in size of the lesions as a reflection of the efficacy of the postoperative EGFR-TKIs. As the largest diameter of most lesions was less than 1 cm, the definition of evaluable lesion of RECIST 1.1 criteria (1 cm or more in largest diameter) could not be met.[3] Therefore, we expanded the lower limit of the diameter to 4mm, as the thin-slice CT scan can precisely detect the minor changes in size by 2mm. The definition of objective response rate (ORR), complete response (CR), partial response (PR), stable disease (SD), and progressive disease (PD) were identical to RECIST 1.1. In addition, we defined the response rate (RR) of EFGR-TKIs as the ratio of the number of patients/lesions with any reduction to the largest diameter of a lesion to the total number of patients/lesions.

***Subgroup Exploration***

Patients were divided into different subgroups, involving sex (male or female), smoking history (yes or no), co-morbidities (yes or no), EGFR-mutation (exon 21 L858R mutation, exon 19 deletion mutation, or rare EGFR mutations), EGFR-TKI (Gefitinib, Erlotinib, Icotinib, Afatinib or Osimertinib), stage (I-II or III), the number of remaining lesions (1-2 or more than 2), and the diameter of the largest remaining lesion (≥8mm or <8mm).

***Evaluation of Genetic Concordance across lesions***

We measured the consistency of gene mutation between multiple lesions when multiple different lesions (≥2) were resected in the same patient. In patients who underwent multi-lesion genetic testing, the ratio of the number of patients with the same mutation type of all lesions to the total number of patients that received testing is herein referred to as the consistency rate of gene mutations.

***Statistical Analyses***

We used the mean and standard deviation to describe the number and the diameter of remaining lesions of patients after surgery. The median and interquartile range (IQR) was used to present the duration and the interval. To compare the response rate, the Chi-Square test was adopted in this study. A multivariable logistic regression model was created using the covariate which showed differences between sub-groups. A P value<0.05 was considered to be statistically significant. Statistical analysis of all data was performed by software SPSS 23.0 (Chicago, IL).

***Ethics approval and consent to participate***

This study was approved by the ethics committee of The First Affiliated Hospital of Guangzhou Medical University. Considering that the study was a retrospective research, informed consent of all patients was waived by the ethics committee.

**Limitations**

There are several limitations in this study. First, we were unable to perform the histopathologic examination for all remaining GGO lesions, however, a multidisciplinary team of thoracic surgeons and radiologists would judge them to be benign or malignant, and these residual GGO lesions were all underwent observation for at least 3 months, without any shrinkage in CT check-up. Second, a good proportion of the residual GGO lesions were smaller than 10mm in diameter, making them unsuitable for evaluation with RECIST criteria, as a result, we estimated the efficacy of EGFR-TKIs using thin-slice CT to observe the changes in size of lesions. Measurement in changes of sub-centimeter lesions are considered accurate in thin-slice CT with a thickness of less than 1mm. Third, PFS or OS benefit could not be evaluated. However, considering the significantly lower rate of second operation in the treatment group, we cautiously reasoned that postoperative EGFR-TKIs treatment might have a favorable impact on the prognosis. Lastly, this seems a retrospective study with small sample size. However, in consideration of the fact that sMPLC were relatively uncommon and only a few of them received postoperative EGFR-TKIs treatment, this samples size was considerable and the current population represented the largest cohort.

**Some detailed explanations of Fig 1(containing Fig.1a, 1b, 1c and 1d) and Table S3.**

***Fig.1a***

We recorded the changes in size of 134 lesions in 66 patients treated with postoperative EGFR-TKIs, with a mean diameter of 7.03±3.41mm. As illustrated in the waterfall plot (Figure 1a), 32 lesions reduced, 7 lesions increased, and 95 lesions had no change in size. The response rate (RR) were 23.9% (32/134) and 33.3% (22/66) of lesions and patients, respectively.

***Fig.1b***

The drug use was recorded in the 22 patients with reduction in size of lesions, and is shown in Figure 1b. The median interval between surgery and drug initiation was 1 month; 12 patients (54.5%) received postoperative treatment with EGFR-TKIs at 1 month (or less) after their surgery. The median time from the beginning of medication to showing the reduction of the lesions was 3.5 months. During EGFR-TKIs treatment of these 22 patients, most patients had continuous response ranging from 1+ to 31+ months, only 3 developed progression after the reduction of lesions.

***Fig.1c***

Among 144 patients with postoperatively residual GGO lesions, 29 patients underwent surgical resection of multiple lesions (2 or 3) and genetic testing; a total of 19 patients showed non-consistent gene mutation between different lesions (65.5%, 19/29).

It's worth noting that the consistency rate of 19 Del mutation was significantly lower than that of 21 L858R mutation (3.4% vs 31.0%, P=0.03). 16 lesions from 13 of these patients showed wild-type EGFR mutation. Gene mutation types of these 29 patients are illustrated in Figure 1c.

As the results show, among patients with EGFR 21 L858R, 19 deletion and rare mutations, the response rates were 38.9%, 28.6% and 14.3%, respectively. Figure 1c could explain the reason why the response rate was higher in patients with EGFR 21 L858R mutation than these with 19 deletion mutation in this study.

***Fig.1d***

We observed the proportion of patients who underwent resection again on residual lesions after primary surgery, that was the second surgery rate. (as described in Figure 1d) The second surgery rate of patients was significantly lower in the EGFR-TKIs treatment group compared to the control group (3.0% vs. 18.2%, P=0.004).

***Table S3***

As shown in Table S3, a multivariable logistic regression model was created using the following covariates: The number of residual malignant GGO lesions, the diameter of the largest unresected GGO lesion, and the patients with different stages (stage I/II or stage III). These covariates were chosen based on the results of Chi-Square test. When considering the characteristics of patients, the efficacy of EGFR-TKIs was associated with a greater number of residual lesions (OR=4.33, P<0.01), larger diameter of the maximal residual lesion (OR=9.07, P<0.01), and higher stage of primary lesions (OR=4.46, P=0.02). In addition, in terms of the characteristics of lesions, lesions with mixed component (OR=2.37, P=0.04) and larger diameter of residual lesions (OR=4.18, P<0.01), were independent predictors for EGFR-TKIs efficacy.

In brief, for sMPLC patients with EGFR mutations, postoperative EGFR-TKIs therapy showed activity on unresected GGO lesions. Furthermore, for these patients there was significant benefit if they presented with TNM stage III, had more than 2 remaining lesions, mixed component lesions, or the diameter of residual lesions ≥8mm. These were all independent predictors associated with the efficacy of EGFR-TKIs.

**References:**

1. Detterbeck FC, Franklin WA, Nicholson AG, Girard N, Arenberg DA, Travis WD, et al. The IASLC Lung Cancer Staging Project: Background Data and Proposed Criteria to Distinguish Separate Primary Lung Cancers from Metastatic Foci in Patients with Two Lung Tumors in the Forthcoming Eighth Edition of the TNM Classification for Lung Cancer. J Thorac Oncol **11,** 651-665 (2016).

2. Goldstraw P, Chansky K, Crowley J, Rami-Porta R, Asamura H, Eberhardt WE, et al. The IASLC Lung Cancer Staging Project: Proposals for Revision of the TNM Stage Groupings in the Forthcoming (Eighth) Edition of the TNM Classification for Lung Cancer. J Thorac Oncol **11,** 39-51 (2016).

3. Eisenhauer EA, Therasse P, Bogaerts J, Schwartz LH, Sargent D, Ford R, et al. New response evaluation criteria in solid tumours: revised RECIST guideline (version 1.1). Eur J Cancer **45,** 228-247 (2009).
